# Sibship Size and Gendered Resource Dilution in Different Societal Contexts

**DOI:** 10.1371/journal.pone.0160953

**Published:** 2016-08-25

**Authors:** Matthijs Kalmijn, Herman G. van de Werfhorst

**Affiliations:** Department of Sociology, University of Amsterdam, Amsterdam, the Netherlands; University of Reading, UNITED KINGDOM

## Abstract

Resource dilution theory hypothesizes that children’s educational attainment suffers from being raised with many siblings, as the parental resources have to be shared with more children. Based on economic and cultural theories, we hypothesize that resource dilution is gendered: especially a larger number of brothers is harmful to a person’s educational attainment. Using the Survey of Health, Ageing and Retirement in Europe, covering 18 European countries, we show that the number of brothers is more negatively related with the odds of obtaining a college degree than the number of sisters. This holds particularly for women. However, this pattern is weaker in countries that are known to have a more gender-egalitarian climate.

## Introduction

Many studies have found a negative impact of the number of siblings on children’s educational achievement and attainment. Children’s cognitive development and educational attainment suffer from growing up with larger numbers of siblings because the resources parents offer to their children have to be shared with more offspring [1–8], and because the cognitive climate deteriorates if new children enter the family [9,10]. More recent instrumental-variable models have partly replicated these results, in particular when relying on the exogenous increase in the number of children that results from having only prior sons or only prior daughters in the family [11]. Other instrumental-variable analyses have been less supportive but these relied on twin births, a special case which is neither representative nor comparable to consecutive births [12–14].

Most of the contemporary literature has assumed that the gender of siblings is not important for understanding how family size affects educational and cognitive outcomes. Theoretically, however, there are good reasons to believe that gender matters. If siblings compete for parental resources, and if parents have a gender bias in the way they invest in children, one would expect that for women, the number of brothers will have a more negative effect on their educational outcomes than the number of sisters. Only a handful of studies have investigated the separate influence of the number of brothers and sisters but the evidence has not been very supportive [4,15–18]. Because of these ‘disappointing’ research findings, the hypothesis of gender-specific sibsize effects has been abandoned.

We examine the gender configuration of siblings again by using a comparative design. Using data from three waves of the Survey of Health, Ageing and Retirement in Europe (SHARE), we examine the separate influences of the number of brothers and sisters on the college completion of men and women. We not only provide new evidence in a field with mixed findings, we also examine cross-national differences in the effects of brothers and sisters. This comparison allows us to examine if effects of the number of children on schooling outcomes vary across societies with different gender role regimes. In societies with high levels of gender inequality, parents will have more traditional views on the economic roles of their sons and daughters and daughters will benefit less from schooling than sons when they are adult. As a result, one would expect that parents in these societies invest more heavily in the schooling of their sons than of their daughters. In other words, the impact of siblings’ gender configurations will be stronger in more traditional contexts than in more egalitarian contexts. We test this idea for 18 European countries ranging from very egalitarian societies such as Sweden and Denmark, to societies with more traditional gender roles, such as Greece, Poland, and the Czech Republic [19]. We examine the comparative question in three ways, by estimating cross-level interactions in logit models on the pooled data, by using a graphical representation of effects in each country, and by estimating meta-regression models on the country-specific regression coefficients.

## Background and Hypotheses

Building on standard resource dilution theory, we expect that children experience a disadvantage from being raised in large families. If parents have to share their resources among more children, each child will receive fewer resources than in families with fewer children. These resources can be of different kinds, and could include financial means to pay for college, time spent on helping children with homework, and taking children out to museums [5,20]. Knowing that financial, cultural, and social resources are crucial for children’s educational attainment, and that these resources are typically finite, having to share resources will lead to a disadvantage for children who have many siblings. In some cases, parental investments are not rivalrous. A mother can read a book to her children simultaneously and a father can talk about the importance of higher education during a family meal. In these cases, resources are not spread out more thinly in larger families. More subtle effects may be operating as well, for instance, of birth order and birth spacing [6,21]. We leave these aside in the present paper since exploratory analyses suggested that these do not interact with the gender of siblings.

Several theories suggest that the gender composition of a family may play a role in children’s educational opportunities and cognitive development. Economically, men are assumed to reap higher benefits from education in the labor market due to household specialization within their own established families. Because parents may want to maximize future earnings of their children, they may be more likely to invest in sons’ than in daughters’ schooling [15,22]. Along similar lines, parents may place more emphasis on the marriage propects for their daughters rather than on their daughters’ own economic future. Similar predictions can be made from a cultural gender role perspective. Traditional gender norms will induce families to have a preference for son’s socioeconomic advancement whereas the advancement of daughters will be encouraged via marriage [23]. Hence, there are both economic and cultural reasons why parents may encourage sons more than daughters to go to college.

By itself this does not imply that daughters are negatively affected by having brothers. Gender preferences only imply that daughters will have fewer educational opportunities in a family than sons. This main gender effect is well-known and not of interest here. Our focus is on the gender effect *of a child’s siblings*. To develop hypotheses about this, we need to return to the argument on resources. The financial and cultural resources that parents have available are competitive: the time that parents have to help one child in school cannot be spent on another child–unless they are very close in age–and the money that parents have to pay for schooling can only be spent once. Given that there is a preference for sons, parental resources will not be distributed equally among sons and daughters. As a consequence, a daughter will be disadvantaged if she has a brother, compared to the situation where she only has a sister. In the latter case, each sister will get ‘half’ the parents’ resources, in the former case, the sister will get less than half. By the same logic, a brother will be advantaged if he has a sister compared to the situation where he has a brother. In the latter case he has to share with his brother, in the former case, he has a ‘weak’ competitor for parental resources. In other words, both sons and daughters will be affected most strongly by having brothers and less by having sisters. We call this the hypothesis of *gendered resource dilution*.

Only a few studies have examined the gendered resource dilution hypothesis and the ones that do provide negative or inconclusive findings. The influence of the number of brothers and the number of sisters have been studied, on financial arrangements, including whether parents pay for college, whether students have side jobs, and whether students have a study loan [18]. This study found support for the hypothesis because brothers (not sisters) appeared to be a ‘liability’ for the resources available to siblings. A later study distinguished the number of brothers and sisters in a study on academic achievement, and found that the number of brothers was more negatively associated to school grades than the number of sisters [24]. Also the time parents spend per child on child rearing activities is dependent on the number of children in the family, with equally negative effects of the number of sons and daughters [25]. These last two studies did, however, not analyze these effects separately for sons and daughters. Studies that examined educational attainment as an outcome present mixed results. One study found that sisters benefited from a larger number of brothers [26] and two studies found no significant effect [15,27]. Finally, a recent German study which focused on the completion of tertiary education, found no negative effect of the total number of brothers but did find negative effects of the number of *older* brothers [4]. This study did not examine the educational attainment of men, however.

Some authors have presented alternative hypotheses about the gender composition of a family. The gender configuration in families is said to determine how siblings operate as reference groups for their own educational aspirations [15]. A masculine culture may spill over to sisters, improving women’s educational performance. From this perspective, women’s orientation to education may be positively affected by having brothers, at least to the extent that education (or achievement more generally) can be considered a masculine trait [28]. This reference group idea leads to the opposite hypothesis as the gendered resource dilution idea and is therefore difficult to test directly.

The notion that families prefer to invest in sons’ human capital accumulation over daughters’ was developed in a time when household specialization between husbands and wives was common, the gender earnings gap was large, and gender norms were more traditional. However, one can question the adequacy of the line of reasoning in societies where gender relations are more egalitarian. It is plausible that gendered resource dilution is not equally found in more gender egalitarian countries compared to countries with a more conservative gender climate. Our European data enable us to study such cross-national differences in-depth. European countries differ significantly with regard to gender climate. How do these country differences impact the gendered resource dilution in families?

It is well-known that countries differ significantly in terms of gender inequality in the labor market and in education, and that these country differences can be explained by differences in social policies, gender ideology and overall levels of wage inequality. Social policy is strongly determining the extent to which households conform to the male breadwinner model [29–31], although the gender gap in earnings seems to be more strongly driven by a compressed wage structure than by family policies alone [32]. Household tasks are more evenly distributed between husbands and wives in households where the wives work fulltime [33], implying that policies that increase female labor force participation result in a more egalitarian division of tasks at home. Furthermore, gender differences in academic achievement are smaller in societies with more gender-egalitarian labor markets and gender-egalitarian ideology [34,35]. The under-representation of women in the high-demand engineering fields is smaller, and women’s college degree attainment is higher in societies with a more gender-egalitarian culture [36]. Based on these differences, we expect that the gendered resource dilution hypothesis is more strongly supported in societies with a more conservative gender climate than in societies with a more gender-egalitarian climate.

## Data, Methods, and Variables

We use data from the *Survey of Health*, *Ageing and Retirement in Europe* (SHARE, http://www.share-project.org from where we accessed the data; interested parties can access the data using the same methods). The questionnaire survey SHARE was conducted in 18 European countries in five waves (2004/2005, 2006/2007, 2008/2009, 2011/2012, 2013). Although the SHARE is a panel survey, in each wave, large refreshment samples were added. These refreshment samples were either new birth cohorts for participating countries or additional countries. Questionnaires were similar across waves except in the third, which was on a special topic (life histories) and which is not used here. We use all data from the first wave and all the ‘new’ cases from waves 2, 4, and 5. We do not utilize the panel nature of the data. We selected so-called ‘family respondents,’ these were the respondents who had to answer the questions about the children. Respondents answered questions on four children in the first and second wave and on all children in the fourth and fifth wave. For families with more than four children, the children who lived closest were targeted. Since the SHARE is based on a sample of the population aged 50+, many respondents have adult children. The total number of (family) respondents in the data is 57,375. Data from Luxembourg and Israel are not used. This sample yields data on 132,209 children.

The data were converted to a file with children as the units of analysis. We limit this sample to children who were at least 25 years old so that they can be assumed to have completed schooling. We further limited the sample to children who were born after World War II in order to reduce the historical heterogeneity of the sample. Finally, we limit the sample to children who had at least one sibling because in one-child families, the gender of the sibship is not defined. After making these selections, we have data on 98,244 children who belong to 41,037 parents. The design was approved by the Ethics Advisory Board of the Amsterdam Institute for Social Science Research, Faculty of Social and Behavioral Sciences, University of Amsterdam,

Our dependent variable is whether or not the child completed a college or university degree (ISCED category 5 and 6) versus a lower level of education (including incomplete college). We focus on this dichotomy because the decision to go to college is probably most sensitive to competition among siblings. It should be noted that the data do not permit to study college enrollment separately from completion; only completed qualification levels are available. The data are analyzed with logit models where the adult children are units. The standard errors are corrected for the clustering of children in families. All analyses are presented separately for women (daughters) and men (sons).

The measurement of siblings is based on reports of the parent (i.e., the primary respondent). We test our hypotheses in a series of models, with different measures for family configurations as independent variables. Model 1 contains a simple measure for sibship size (sibsize, for short). Model 2 replaces this measure with two measures: the number of brothers and the number of sisters:

$$\ln\;\left(\frac{P_{i}}{1-P_{i}}\right)=a_{0}+a_{1}B_{i}+a_{2}S_{i}+e_{i}$$ [1]

where B_i_ is the number of brothers and S_i_ is the number of sisters. A test of our hypothesis is obtained by examining if the effect of the number of brothers and sisters is different. This statistical model gets close to the behavioral model underlying gendered resource dilution, as it tells us how educational decision making is affected by having another competing brother *instead of* another competing sister. The following model is estimated:

$$\ln\;\left(\frac{P_{i}}{1-P_{i}}\right)=b_{0}+b_{1}(B_{i}+S_{i})+b_{2}(B_{i}-S_{i})/2+e_{i}$$ [2]

When comparing Model 2 and 3, we see that a_1_ = b_1_ + ½ b_2_ and a_2_ = b_1_ –½ b_2_ so that b_2_ = a_1_ –a_2_. In words, b_2_ tests the difference in the additive net effects of brothers (a_1_) and sisters (a_2_).

Note that we treat the gender composition of the siblings as exogenous. While family size can be predicted by the gender composition of the first siblings (because two same-sex children increases the chance for parents to get another child; [37,38]), this is not the case for the gender composition of siblings. Furthermore, there is no correlation between parents’ educational level and the proportion of brothers in the children’s generation in our data (*r* = -0.003 for father’s education and *r =* -.009 for mother’s education). This shows that the gender composition of siblings is unrelated to socioeconomic background, unlike the number of siblings itself, which is correlated -.14 with father’s education and -.16 with mother’s education.

As control variables, we use a simple indicator for birth order in all models, i.e., whether the child was the oldest child or not [6]. We also include a variable indicating if the child was a stepchild, an adopted child or a foster child (i.e., a stepchild of either the respondent or the partner). We include the completed educational level of the parents. To construct father’s and mother’s education, we use information on the partner of the family respondent (if the partner participated) and information reported by the respondent on the former spouse in case the respondent is divorced, separated or widowed. In 22.8% of the cases, the information on the spouse is missing. When constructing father’s and mother’s education using the respondent’s and the spouse’s education, this leads to 12.8% missings on father’s education and 10.6% missings on mother’s education. These values are imputed at country-specific mean levels and dummy-variables are included indicating that the value was imputed. Since we are considering a control variable here, we chose for this relatively simple and straightforward imputation method rather than for a more advanced and more complicated multiple imputation procedure. Similar to most existing studies, parental education is included in linear form, and coded in years of education. The years of education variable is recoded from five categories of the UNESCO-ISCED97 classification using norms provided by the SHARE data project (primary education ISCED 1 or less: 6 years; lower secondary education ISCED 2: 9 years; upper secondary education ISCED 3: 12 years, post-secondary non-degree level education ISCED 4: 14 years, and tertiary degree-level education or more ISCED 5–6: 16 years). The correlation between father’s and mother’s education is *r* = .61, which is as one would expect.

We also included household income of the parents. Total household income is available in all waves. Missing values are imputed by SHARE and used here as well. Note that the income information applies to the present situation and not to the time in which the specific child was raised. We solved this problem in part by adjusting the income for the age of the family respondent. More specifically, we created a rank score within age-country-wave combinations (where age was coded into five groups). We realize that there are more subtle adjustments possible but we doubt that these will affect the main income effects in a significant fashion. We think that age/cohort is a major source of variation in income and after correcting for this, we find substantial income effects on children’s schooling. We note that including the income variable to the model does reduce the effect of the number of siblings it does not affect the differential in the effect of brothers vis-à-vis sisters.Birth cohort is included as a linear variable running from 1–4 (1: 1945–1959, 1960–1969, 1970–1979; and 1980–1988) to adjust for the historical increase in higher education. Country differences are adjusted with country dummies. We include interactions of country and (linear) cohort because the process of educational expansion has not been the same in each country. This last set of controls is quite large and is therefore not printed in the tables. The main effect of cohort applies to the reference country which is Germany. Descriptive statistics of the used variables are displayed in Table 1.

**Table 1:** Descriptive statistics of independent variables.

	mean	sd	min	max	count
Number of sibs	2.093	1.351	1	9	98244
Number of brothers	1.055	1.001	0	9	98244
Number of sisters	1.038	1.001	0	8	98244
Oldest child	0.413		0	1	98244
Non-bio child	0.100		0	1	98244
Relative hh income	0.508	0.286	0.001	1.000	98244
Father's education	10.731	3.389	6	16	98244
Father’s education missing	0.119		0	1	98244
Mother’s education	10.198	3.348	6	16	98244
Mother’s education missing	0.097		0	1	98244
Birth cohort	2.521	0.946	1	4	97394
Wave 2 vs 1	0.153		0	1	98244
Wave 4 vs 1	0.392		0	1	98244
Wave 5 vs 1	0.169		0	1	98244

Note: Families with 2 or more children. Source: SHARE waves 1, 2, 4, and 5 (only new respondents).

We examine the role of the gendered societal context by ranking countries using the *Gender Inequality Index* of the United Nations (GII) [39]. This index combines information about women’s relative employment, education, mortality, political representation, and adolescent fertility. We first present a graphic view of national differences by plotting the effects of the number of brothers and sisters (from Model 2) for each country against the level of gender inequality in each country. Secondly, we use models with cross-level interactions based on the ranked GII, using the z-score of the ranked GII index. These estimates are based on clustered logit models because the number of societal contexts is rather small for applying true multilevel models [40]. Third, to test the interaction in a more formal way, we apply meta-regression where the countries are the units of analysis, the regression coefficients in each country are the outcome variables, and the gender index is the independent variable. In these models, the data are weighted by the (inverse of the) standard errors of the regression coefficients [41]. We believe the strength of the evidence lies in the combined use of multilevel models, graphic methods, and meta-regression at the aggregate level.

## Results

We start with a descriptive analysis. Table 2 shows the distribution of families across the different gender configurations, by sibship size. We see that the gender configuration follows what one would expect given that having a son or daughter is more or less random. In larger families there is more fluctuation but this is not in a specific direction. For some family sizes, there are more majority-son families than majority-daughter families, for other family sizes, it is the other way around. Fortunately, these differences are small.

**Table 2:** Sex composition by family size.

	All sons	Majority sons	Equal	Majority daughters	All daughters	Total
	Row % / n	Row % / n	Row % / n	Row % / n	Row % / n	Row % / n
1 child	49.9	0.0	0.0	0.0	50.1	100.0
	4897	0	0	0	4924	9821
2 children	24.3	0.0	52.8	0.0	23.0	100.0
	10206	0	22195	0	9661	42062
3 children	13.5	37.2	0.0	36.4	12.9	100.0
	3913	10761	0	10553	3739	28966
4 children	7.2	25.1	37.0	23.7	7.0	100.0
	1052	3665	5395	3459	1029	14600
5 children	3.5	46.7	0.0	46.4	3.5	100.0
	223	2999	0	2980	224	6426
6 children	2.7	34.4	28.0	32.7	2.2	100.0
	86	1098	893	1044	69	3190
7 children	1.6	47.1	0.0	50.8	0.5	100.0
	25	758	0	817	8	1608
8 children	1.8	35.7	27.7	33.9	0.9	100.0
	15	302	234	287	8	846
9 children	0.0	51.9	0.0	47.0	1.1	100.0
	0	182	0	165	4	351
10 children	0.5	24.6	33.3	41.5	0.0	100.0
	1	48	65	81	0	195
Total	18.9	18.3	26.6	17.9	18.2	100.0
	20418	19813	28782	19386	19666	108065

Source: SHARE waves 1, 2, 4, and 5 (only new respondents).

**Fig 1:**
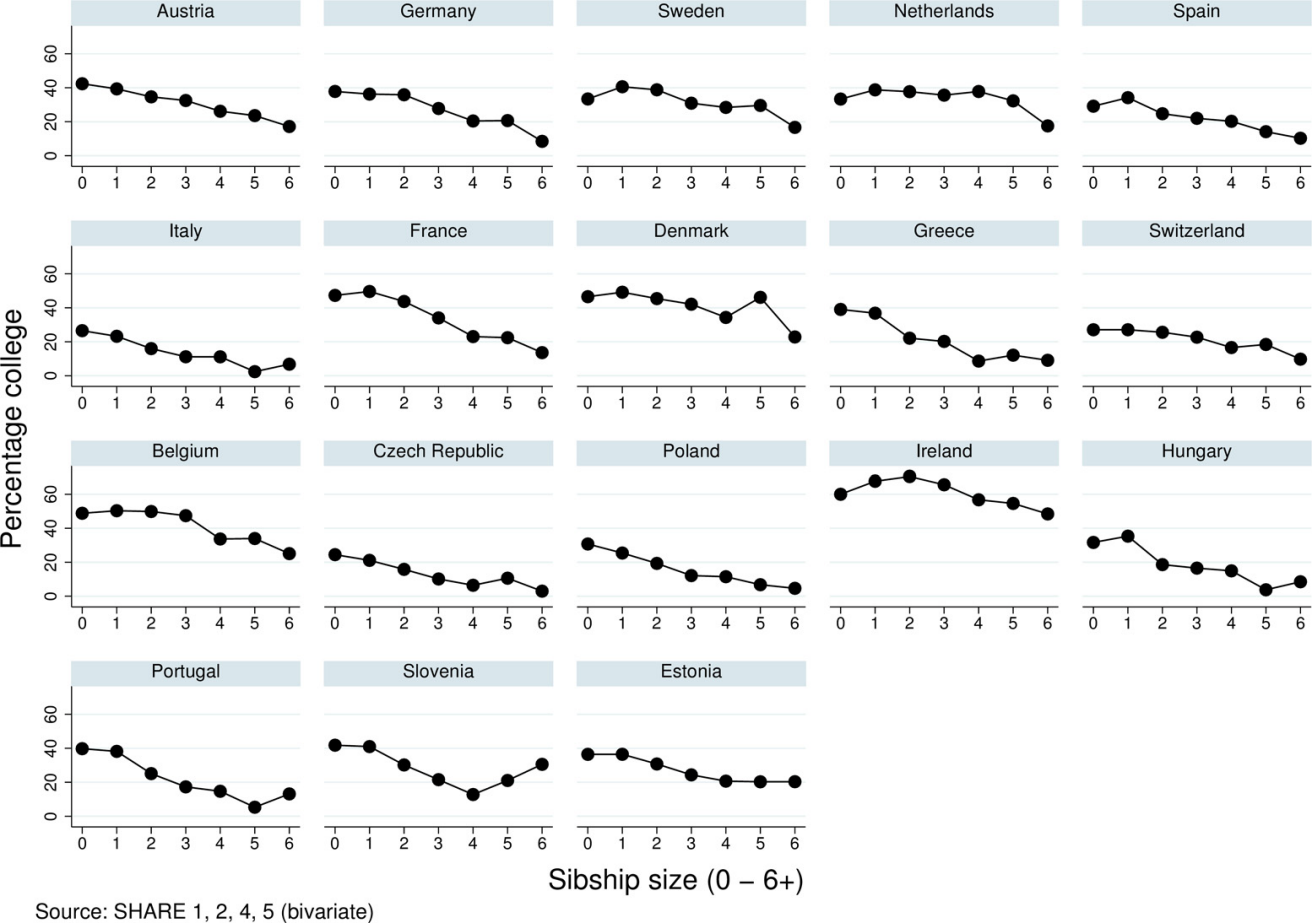
College degree by sibship size in Europe.

In Fig 1, we present the percentage of sons and daughters who complete college broken down by sibship size. The graphs show that, in most countries, the chances to obtain a college degree are negatively correlated to family size, just as standard resource dilution theory predicts. In a few countries, the pattern is rather flat for the more common family sizes. In some countries singletons appear to do worse but this may be due to selection effects (i.e., parity progression is dependent on the health of the prior child). Part of these effects will be biased by cohort and parental education; these are simple bivariate plots. Lower educated parents have more children and sibship sizes decline across cohorts, this will bias the effects in an upward direction.

Table 3 shows the results of the logistic regression models predicting college attainment for daughters. Model 1 replicates the standard resource dilution model. In line with this model, we find a significant negative effect of family size. For each additional child in the family, daughters’ odds to have a college degree decline with 19% (i.e., 1 –*e* ^-.21^). In Model 2, we control for family socioecoeconomic status (parents’ education and household income). These variables have the strong expected relationship to daughters’ educational attainment, with the odds of completing college increasing with 0.16 with an additional year of both father’s and mother’s education, and an increase of the odds of 0.69 across the theoretically maximum range of relative household incomes. The dummy variables representing imputed cases are, reassuringly, not significantly associated with daughters’ college completion. Inclusion of the parental indicators decrease the sibship size effect, which is still significant and sizeable at b = -0.138 (representing a decline of the odds of a college degree with 13%).

**Table 3:** Logistic regression college education for women: Coefficients and robust standard errors.

	Model 1	Model 2	Model 3	Model 4	Model 5	Model 6
College vs lower						
Number of sibs	-.210*	-.138*		-.139*		-.141*
	(.011)	(.011)		(.011)		(.011)
x Gender inequality (z)						-.032*
						(.012)
Number of brothers			-.152*		-.156*	
			(.014)		(.014)	
x Gender inequality (z)					-.046*	
					(.014)	
Number of sisters			-.125*		-.126*	
			(.014)		(.014)	
x Gender inequality (z)					-.018	
					(.015)	
Brothers vs sisters				-.027		-.030^~^
				(.017)		(.018)
x Gender inequality (z)						-.028
						(.018)
Oldest child	.159*	.067*	.067*	.067*	.067*	.067*
	(.019)	(.021)	(.021)	(.021)	(.021)	(.021)
Non-bio child	-.147*	-.410*	-.409*	-.409*	-.410*	-.410*
	(.039)	(.042)	(.042)	(.042)	(.042)	(.042)
Relative hh income		.689*	.689*	.689*	.693*	.693*
		(.045)	(.045)	(.045)	(.045)	(.045)
Fathers education		.155*	.155*	.155*	.155*	.155*
		(.005)	(.005)	(.005)	(.005)	(.005)
Info missing		.050	.050	.050	.050	.050
		(.036)	(.036)	(.036)	(.036)	(.036)
Mothers education		.161*	.161*	.161*	.161*	.161*
		(.005)	(.005)	(.005)	(.005)	(.005)
Info missing		-.047	-.046	-.046	-.047	-.047
		(.040)	(.040)	(.040)	(.040)	(.040)
Birth cohort	.012	-.101*	-.100*	-.100*	-.097*	-.098*
	(.040)	(.042)	(.042)	(.042)	(.042)	(.042)
Wave 2 vs 1	-.013	.006	.007	.007	.006	.006
	(.045)	(.048)	(.048)	(.048)	(.048)	(.048)
Wave 4 vs 1	-.002	-.027	-.028	-.028	-.029	-.029
	(.038)	(.040)	(.040)	(.040)	(.040)	(.040)
Wave 5 vs 1	.114*	.059	.058	.058	.057	.057
	(.039)	(.041)	(.041)	(.041)	(.041)	(.041)
Persons	48205	48205	48205	48205	48205	48205
Log likelihood	-29051	-25867	-25866	-25866	-25859	-25859

Note: Controlled for country dummies and interaction of country and cohort. Source: SHARE waves 1, 2, 4, and 5 (only new respondents).

~ p < .10

* p < .05.

In Model 3, we split the family size variable into the number of brothers and the number of sisters, offering a first test of our gendered resource dilution hypothesis. The number of brothers in Model 3 has a more negative effect than the number of sisters. The difference is not small: the effect of brothers is 22% larger than the effect of sisters. When we test the difference between these effects in Model 4, we see an insignificant result. In Model 5, we add interaction terms with the ranked gender inequality index, testing for non-linearities in the gender composition variables. It should be noted that there is no main effect of the gender inequality index because all models include country fixed effects. The model shows that the negative effect of brothers gets significantly stronger in more gender-unequal societies. For every standard deviation on the gender inequality indicator, the effect of number of brothers varies by a third of the effect size (-0.156 versus -0.202). The strength of the effect of number of sisters on daughters’ college attainment does not vary between countries of different levels of gender inequality. Hence, in line with our gendered resource dilution hypothesis, brothers are a greater liability than sisters for women’s educational attainment. Model 6 tests for the statistical difference of the slopes of brothers and sisters and the interaction with the gender inequality index. The main effect is very similar to the model without interactions, and the interaction term is not significant.

We now turn to the results for sons in Table 4. We again see a negative effect of family size. For each additional sibling in the family, the odds of going to college decline by 18% (i.e., 1 –*e* ^-.195^). The effect is similar to what it is for daughters, suggesting already that siblings do not compete more for daughters than for sons. Model 2 controls for family socioeconomic status, and the reduction of the sibship size effect is also similar to what it was for daughters (and still statistically significant). Model 3 shows that the negative effects of brothers and sisters are very similar for men’s college attainment, and model 4 confirms that the difference is small and not significant. Model 5 shows that the number of siblings, both brothers and sisters, is more harmful to sons’ likelihood to obtain a college degree in more gender-inegalitarian societies. Nevertheless, for brothers the interaction effect with gender inequality is 0.37 of the main effect (-0.049/-0.133), while for sisters it is 0.30 (-0.041/-0.137), pointing to slightly stronger dependence of the brothers coefficient on the gender climate than of the sisters coefficient. Model 6 shows, however, that the number of brothers and sisters have similar effects on sons’ college attainment independent of the level of gender inequality in a society. So the evidence in favor of the the gendered resource dilution hypothesis for men is limited, according to which sisters would be expected to be of lesser influence on educational outcomes than brothers.

**Table 4:** Logistic regression college education for men: Coefficients and robust standard errors.

	Model 1	Model 2	Model 3	Model 4	Model 5	Model 6
College vs lower						
Number of sibs	-.195*	-.127*		-.127*		-.135*
	(.011)	(.011)		(.011)		(.012)
x Gender inequality (z)						-.045*
						(.012)
Number of brothers			-.124*		-.133*	
			(.015)		(.015)	
x Gender inequality (z)					-.049*	
					(.015)	
Number of sisters			-.130*		-.137*	
			(.014)		(.015)	
x Gender inequality (z)					-.041*	
					(.015)	
Brothers vs sisters				.007		.004
				(.018)		(.018)
x Gender inequality (z)						-.009
						(.018)
Oldest child	.180*	.091*	.091*	.091*	.091*	.091*
	(.020)	(.021)	(.021)	(.021)	(.021)	(.021)
Non-bio child	-.247*	-.504*	-.504*	-.504*	-.508*	-.508*
	(.042)	(.043)	(.043)	(.043)	(.044)	(.044)
Relative hh income		.667*	.666*	.666*	.670*	.670*
		(.046)	(.046)	(.046)	(.046)	(.046)
Fathers education		.168*	.168*	.168*	.168*	.168*
		(.005)	(.005)	(.005)	(.005)	(.005)
Info missing		-.084*	-.083*	-.083*	-.084*	-.084*
		(.037)	(.037)	(.037)	(.037)	(.037)
Mothers education		.134*	.134*	.134*	.134*	.134*
		(.005)	(.005)	(.005)	(.005)	(.005)
Info missing		-.041	-.041	-.041	-.043	-.043
		(.040)	(.040)	(.040)	(.040)	(.040)
Birth cohort	-.121*	-.233*	-.233*	-.233*	-.229*	-.229*
	(.039)	(.040)	(.040)	(.040)	(.040)	(.040)
Wave 2 vs 1	-.094*	-.099*	-.099*	-.099*	-.098*	-.098*
	(.044)	(.046)	(.046)	(.046)	(.046)	(.046)
Wave 4 vs 1	-.032	-.051	-.051	-.051	-.053	-.053
	(.038)	(.040)	(.040)	(.040)	(.040)	(.040)
Wave 5 vs 1	.108*	.067	.067	.067	.066	.066
	(.040)	(.041)	(.041)	(.041)	(.041)	(.041)
Persons	49189	49189	49189	49189	49189	49189
Log likelihood	-28402	-25500	-25500	-25500	-25490	-25490

Note: Controlled for country dummies and interaction of country and cohort. Source: SHARE waves 1, 2, 4, and 5 (only new respondents).

* p < .05.

The control variables have the effects that we would expect. Men and women who are the first-born child have higher odds to go to college. Non-biological brothers and sisters–mostly stepchildren–have a lower odds to go to college.

To further examine the international differences in gendered resource dilution, we employ a meta-regression on the slopes of the number of brothers and sisters, estimated separately by country. Cases (estimates) are weighted by the inverse of its standard error. Table 5 shows the results. In line with the logistic regression models with country fixed effects, the negative effect of the number of brothers gets more negative for women’s college completion rates in more gender-inegalitarian societies (p = 0.057 on N = 18). The effect of number of sisters on women’s attainment is hardly related to the gender climate in a society. The number of brothers is also harmful to men’s college attainment in more gender-inegalitarian societies, while the effect of gender inequality on the slope for sisters is not significant but only marginally smaller. So, clearly, brothers are a liability for college attainment, particularly for women, and particularly in gender-inegalitarian contexts.

**Table 5:** Meta-regression for men and women: Coefficients, standard errors in brackets, and p-values in square brackets.

	Effect brothers for women	Effect sisters for women	Effect brothers for men	Effect sisters for men
Gender inequality (z)	-.048^~^	-.014	-.039^*^	-.032
	(.023)	(.020)	(.015)	(.019)
	[.057]	[.497]	[.018]	[.103]
Constant	-.167^*^	-.130^*^	-.138^*^	-.151^*^
	(.024)	(.020)	(.015)	(.019)
	[.000]	[.000]	[.000]	[.000]
Countries	18	18	18	18
F-test	4.20	.48	6.99	2.99

Source: SHARE waves 1, 2, 4, and 5 (only new respondents).

~ p < .10

* p < .05.

**Fig 2:**
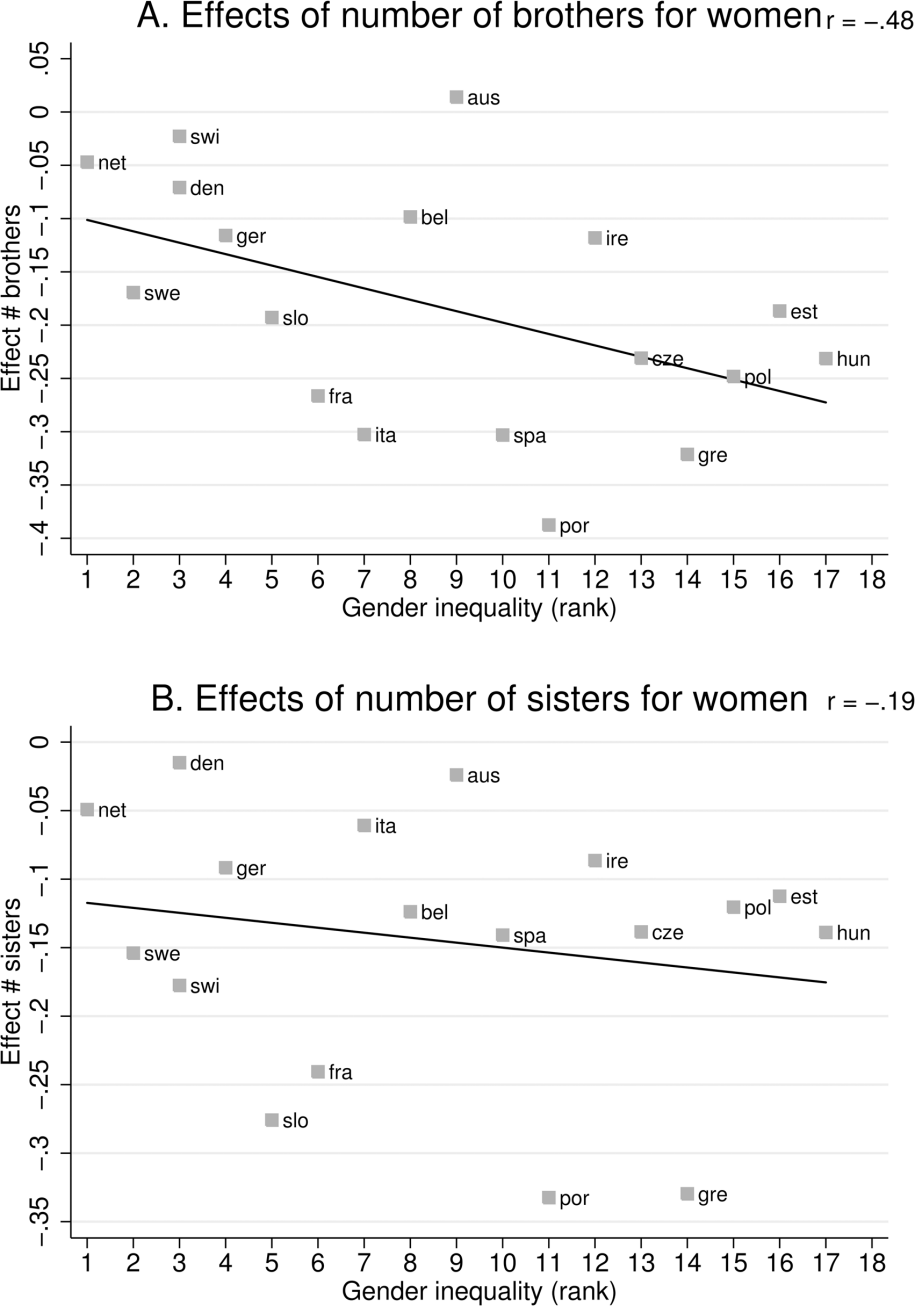
Effect of brothers (A) and sisters (B) on women’s college education by gender inequality index.

To further explore the role of cross-national differences in the effects of the number of brothers on women’s odds of completing college, we examine the countries one-by-one and use a graphic presentation. We estimated Model 3 in each country and plot the unstandardized effect sizes of the number of brothers and sisters for women’s college attainment agains the gender inequality score at the macro-level. The results are presented in Fig 2A for brothers and 2b for sisters. The negative effect of the number of brothers tends to be stronger in countries with more gender inequality (Fig 2A). The scatterplot for the effects of sisters appears less systematic. The the negative effect of the number of sisters is less dependent on gender inequality (Fig 2B). A simple correlation for the two scatterplots show that the effect of brothers is strongly related to gender inequality (r = -.48), whereas the effect of the number of sisters is weakly related (r = -.19). Note that these effects were obtained from the same country-specific model.

## Conclusion

This paper argued that the dilution of resources, which is believed to form the basis of the negative association between level of educational attainment and family size, is gendered. Families have to share resources in large families, but having siblings is particularly harmful for a child’s likelihood to enter and complete college if those siblings are brothers. Resource dilution is therefore not gender-neutral, that is our main hypothesis. Our analysis of adult children in 18 European countries shows that the evidence for this effect depends on the societal context as well as on gender itself.

For women, we find more negative effects of brothers than of sisters. Moreover, the negative effect of brothers is much stronger in societies with unequal gender climates. The (smaller) negative effect of sisters on women’s college completion is not dependent on the level of gender inequality in society. The reasons for this effect were not studied in this paper but we believe that both rational (economic) and normative (cultural) arguments play a role. In highly gender-inegalitarian societies, parents will be focused on the socioeconomic status of their sons and will believe that the success of their daughters mainly depends on marriage. Similarly, if parents want to maximize their children’s earnings, they would be better off investing in sons since the payoff for men later will be higher, but again, only in gender unequal societies.

While these findings are supportive of our general framework, the results for men require more thought. For men, we hardly find stronger negative effects of brothers than of sisters, while the gendered resource dilution theory would also predict stronger negative effects of brothers. We do find a modestly stronger dependence of the brothers effect on the gender climate in a society.

These findings illustrate that the context is highly influential on how children benefit from the resources that are available in the family. Context in the form of the gender configuration in the family impacts men and women differently, while the relevance of the family context itself depends on the gender climate in the country of residence. Moreover, the gender climate effects may explain why earlier studies have been inconclusive on how the gender configuration in families matters. In some countries, there is no gender-specific effect, in other countries, there is more evidence for such an effect. In our view, the hypothesis of gendered resource dilution needs to be reinstated as an important view on how gender inequality is linked to the family of origin.

In a more general way, the link between sibling’s gender configuration and children’s educational outcomes is relevant because it provides new evidence on gender inequality in contemporary western societies. Most studies on gender inequality focus on differences in ‘outcomes’ between men and women, for instance, in terms of wages, occupational choices, political power, and so on [42–45]. One of the challenges in these studies has been to assess to what extent gender differences arise from differential treatment on the one hand, and differences in men and women’s own values, motivations, and personality traits on the other. Even relatively straightforward research designs that statistically control for human capital in wage equations yield indirect results, with net gender effects that can both be overestimates and underestimates of differential treatment based on gender. Studying gender inequality among siblings does not provide solutions to this problem either as differences between sons and daughters in educational attainment simply reflect gender differences in education in society at large. Our analysis offers an alternative and more subtle piece of evidence that there is differential treatment of men and women.

We end with some methodological caveats. There has been debate about whether sibsize effects are truly causal. Several authors have argued that there are unmeasured background variables which may render the effect spurious if they would have been included in the model [13,46]. We note that most papers provide an extensive array of background variables that are relevant for children’s schooling and that could be confounders of the effect [5]. It is difficult to imagine that there would be many other powerful predictors of children’s education that are not already captured by these rich background variables. The critical analyses that have been published do not suggest any substantive variables either nor do they develop theoretical perspectives for explaining selection bias. More importantly, it is implausible that the gender-specific sibsize effects are also affected by selection bias. The sex of child is random and there is no association between the proportion of sons and sibsize. Fertility decisions do depend on the mix of genders, but not on the share of sons or daughters itself. For this reason, we think that our central variable of interest is closer to an exogeneous variable than the number of siblings itself.
